# Clinical remission in juvenile idiopathic arthritis after termination of *etanercept*

**DOI:** 10.1007/s00296-012-2468-3

**Published:** 2012-07-21

**Authors:** Jacek Postępski, Katarzyna Kobusińska, Edyta Olesińska, Violetta Osińska, Violetta Opoka-Winiarska

**Affiliations:** 1 Department of Pediatric Lung Diseases and Rheumatology, Medical University of Lublin, Lublin, Poland; 2 Cardiology and Rheumatology with Neonatal Pathology Unit, Department of Pediatrics, District Pediatric Hospital of J. Brudziński, Bydgoszcz, Poland; 3 Department of Pediatric Lung Diseases and Rheumatology, Children’s University Hospital, ul. Chodźki 2, 20-093 Lublin, Poland

**Keywords:** Juvenile idiopathic arthritis, *Etanercept*, Remission, Therapy discontinuation

## Abstract

Biologicals are very effective for inhibiting disease progression in active juvenile idiopathic arthritis (JIA). To date, there have been no recommendations on how and when to stop therapy with TNF inhibitors. Our objective was to analyze characteristics and the disease course of JIA patients who discontinued *etanercept* due to achievement of inactive disease. Data of 39 patients with JIA from two clinical pediatric rheumatology centers in Bydgoszcz and Lublin (Poland) were analyzed retrospectively. All patients discontinued *etanercept* due to a remission on treatment. *Etanercept* was started after a mean 33.7 ± 36 (range 3–137) months of disease. The mean duration of therapy with *etanercept* was 34.7 ± 16.7 (range 6–72) months, with a mean duration of remission on medication 21.3 ± 9.6 (range 4–42) months before withdrawal of *etanercept*. The mean duration of remission after *etanercept* discontinuation was 14.2 ± 12.1 (range of 1–60) months. Only 12/39 (30.8 %) patients did not develop a disease exacerbation until the end of the study. Early flares, that is less than 6 months after termination of *etanercept*, were observed in 15/39 (38.5 %) patients. Twelve (30.8 %) patients restarted *etanercept* after exacerbation—all patients responded satisfactorily. Our data show that *etanercept* discontinuation in a substantial proportion of JIA patients results in early disease exacerbation. In many cases, reintroduction of *etanercept* is needed. Patients, in whom *etanercept* was restarted, responded satisfactorily.

## Introduction

Juvenile idiopathic arthritis (JIA) is the most common rheumatic disease of childhood. The essence of the disease is a chronic autoimmune inflammation. The main therapeutic goal should be to achieve the reduction of inflammation and initial improvement, then arriving at remission of disease.

For many patients to achieve inactive disease is difficult. In spite of treatment applied, in some patients, disease activity is continued into adulthood [1, 2].

Following a better understanding of the pathogenesis of JIA, in the last 20 years, the principles of pharmacotherapy in JIA have changed. Initially, synthetic and now also biological disease-modifying drugs (DMARDs) are the main element of the therapy. Early initiated, intensely combined treatment creates the possibility of achieving inactive disease (ID) status and its remission.

The preliminary criteria for clinical remission, for selected categories of juvenile idiopathic arthritis, were described by Wallace et al. [3] in 2004.

Experience of over a decade of TNF inhibitors use in JIA allowed formulating the criteria for inclusion treatment in JIA. So far, there have been recommendations on how and when we should stop TNF inhibitor therapy. The questions are: how long should one treat the problem biologically? What factors influence the duration of remission after biological treatment? Should biological drug be discontinued suddenly or gradually by reduction of the dose or should one prolong the intervals between doses?

Six-month disease inactivity is required for fulfilling the criteria for inactive disease on medication, and 12 months for the criteria for inactive disease off medications.

## Objective

We analyzed the disease course of JIA patients that discontinued *etanercept* due to achievement of the stage of inactive disease.

## Materials and methods

The data of 39 patients (12—male, 27—female), with JIA from two clinical pediatric rheumatology centers in Lublin and Bydgoszcz (Poland), were analyzed retrospectively. All patients discontinued *etanercept* due to a remission on treatment. Inactive disease was defined according to the preliminary criteria of Wallace et al. [3]. The clinical subtypes of JIA were systemic—7, polyarthritis—14, oligoarthritis—14, enthesitis-related arthritis—3, psoriatic arthritis-1.

Patients were treated with *etanercept* 2 times a week, 0.4 mg per kg of body weight per dose, until the end of therapy. Treatment was terminated abruptly.

## Results

*Etanercept* was started after a mean 33.7 ± 36 (range 3–137) months of disease. The mean duration of therapy with *etanercept* was 34.7 ± 16.7 (range 6–72) months, with a mean duration of remission on medication 21.3 ± 9.6 (range 4–42) months before withdrawal of *etanercept*.

The mean duration of remission after *etanercept* discontinuation was 14.2 ± 12.1 (range 1–60) months. 12/39 (30.8 %) patients, until the end of the study, did not develop a disease exacerbation and remained in long-term remission off medication—a mean 25.4 ± 12 (range 16–60) months. Because of exacerbation of the disease, 24 children required treatment with methotrexate, 5 with cyclosporine A, 1 patient required treatment with hydroxychlorochine. Early flares, that is less than 6 months after termination of *etanercept*, were observed in 15/39 (38.5 %) patients.

Twelve (30.8 %) patients restarted *etanercept* after exacerbation, due to lack of improvement after no biological DMARDs. All patients in whom *etanercept* was reinitiated responded satisfactorily.

Statistical analysis did not show any correlation between gender, age and type of onset, duration of disease before *etanercept* introduction to therapy, duration of treatment with *etanercept* or sustaining remission off medication.

## Discussion

Only in some scarce reports, the duration of *etanercept* therapy was being investigated, and the question how to terminate *etanercept* therapy for inactive disease in JIA was tackled. The groups of patients presented till now and quoted below were less numerous than as we describe it.

The mean duration of *etanercept* therapy in our group of children was 34.7 months. Prince et al. (19 patients) [4] and Pratsidou-Gertsi et al. (11 patients) [5] treated their patients similarly long with *etanercept* whose durations were 35.1 and 36 months, respectively. In Remesal et al. (26 patients) study [6], the mean duration on therapy with *etanercept* was shorter—that is about 19 months.

In a cohort of 483 *etanercept*-treated JIA patients monitored in the British Register [7], a total of 100 (20.7 %) patients discontinued *etanercept*; only 9 due to disease control. The authors report that in their group followed for a median of 2 years (maximum 5 years), the majority (69 %) remain on the drug.

In our patients, the mean time of remission after discontinuation of *etanercept* was 14.2 months, although it should be noted that in as many as 15/39 children (38.5 %), exacerbation occurred in less than 6 months.

Prince et al. [4] analyzed the clinical course of the disease in 19 patients with JIA after discontinuation of *etanercept* because of sustained good clinical response. In ten of them, remission lasted over a median of 0.8 years. It was found that the longer clinical remission off *etanercept* therapy was associated with previous longer clinical remission on *etanercept*. In the remaining 9 patients, exacerbation occurred earlier than 0.8 years. It was noted that in four of them, *etanercept* was withdrawn abruptly. The authors suggest the beneficial effect of prolonged duration of therapy during clinical remission on medication. They also suggest tapering the *etanercept* dose carefully. Prince et al. [4] believes that patients with JIA should meet the criteria of clinical remission on *etanercept* medication for at least of 1.5 years before considering discontinuation of *etanercept*.

Our study did not confirm any correlation with demographic characteristics, clinical symptoms and a course of treatment.

Pratsidou-Gertsi et al. [5] evaluated the course of the disease in 11 patients with JIA (9—polyarthritis, 2—oligoarthritis disease course) who discontinued *etanercept* after achieving remission. The Juvenile Arthritis Disease Activity Score (JADAS) was used to grade the JIA activity at the time of *etanercept* commencement, at discontinuation and at the time of the flare. Patients were assessed in time from 12.25 to 27 months after discontinuation of *etanercept*. The mean duration of treatment with *etanercept* was 36 months. In all patients, flares of the disease occurred. The mean duration of remission after discontinuation of *etanercept* was 3 months. Exacerbation was successfully controlled in 10 patients with methothrexate or methothrexate and cyclosporine and in one child methothrexate in conjunction with *etanercept*.

Our results are in accordance with the mentioned studies. The mean time of *etanercept* treatment was similar and amounted 34.7 months. Almost one-third of patients remained in clinical remission off therapy. The mean duration of remission after *etanercept* withdrawal was 25.4 months, and remission was held up until the end of the study.

Remesal et al. [6] presented interesting results of a retrospective analysis of 26 patients with JIA who discontinued therapy with *etanercept* due to the inactivation of the disease. The clinical subtypes of JIA were: 11 cases of enthesitis-related arthritis, 7-rheumatoid factor-negative polyarthritis cases, 2 cases of systemic JIA with polyarticular involvement, 1 psoriatic arthritis and 1 persistent oligoarthritis. Fourteen patients withdrew from therapy with *etanercept* abruptly, in 12 either by reducing the dose or by increasing the interval between doses. *Etanercept* was restarted on all relapsed patients. The mean treatment duration was 19 ± 8.4 (9.6–38.5) months. Mean duration of inactive disease before *etanercept* discontinuation was 14.7 ± 8.6 (1–36) months. Eighteen cases (69 %) relapsed at a mean of 5.8 ± 5.3 (0.6–15.9) months after drug discontinuation, whereas in the other 8 (31 %) patients, the disease remained inactive for a mean of 21 ± 14.7 (range 5–44.5) months. In 9 patients, after withdrawal of *etanercept*, the disease remained inactive for at least 12 months. In 4 of them, it exacerbated from 1.5 to 4 months later, and the remaining 5 continued in remission for a mean of 17 ± 13 (range 1.1–32.5) months until the end of the study. It was shown that cumulative probability of maintaining the disease in the inactive state for 6 months after treatment with *etanercept* is characteristic of 50 % of patients and for 12 months—of 39 %. There was no significant difference in duration of remission between the groups of patients in whom *etanercept* was discontinued abruptly or the dose was reduced gradually (11 vs. 14 months *p* = 0.48). There was no correlation between the duration of remission on *etanercept* and the duration of the remission after *etanercept* discontinuation. All patients in whom *etanercept* was restarted responded satisfactorily. The authors conclude that majority of patients (69 %) relapsed after *etanercept* discontinuation. The probability of maintaining the disease in asymptomatic stage for 6 months after termination of therapy is 50 %. In contrast to the results of Prince et al. [4], this analysis did not achieve significant correlation between duration of clinical remission on *etanercept* therapy and duration of the remission after discontinuation.

It is interesting that despite the shorter duration of *etanercept* treatment and shorter duration of clinical remission on therapy, in Remesal et al. [6] observation, we found a similar proportion of relapses after *etanercept* treatment (about 69 %).

Incidence of early exacerbations, that is up to 6 months, is varied. In our study group, the exacerbations up to 6 months occurred in 15/39 (38.5 %). In group of Remesal et al. [6] in 18/26 (69 %), remission was maintained in the mean time of 5.8 ± 5.3 months. In the small group presented by Prince et al. [4], this proportion was 4/19 (21.1 %), while in the similar group of Pratsidou-Gertsi et al. [5], the mean duration of the remission off *etanercept* was only 3 months.

In 12/26 patients of Remesal et al. [6], the dose of *etanercept* was reduced gradually. There was no exacerbation of the disease until complete withdrawal, which might suggest that low doses of *etanercept* may be effective to maintain remission. Prince et al. [5] also suggest gradual reduction of the dose of *etanercept*. The authors pointed out that early exacerbations, that is less than 0.8 years, were observed in 4/19 (21 %) children whose therapy was finished abruptly. Our observations and the observations of other authors show a high proportion of early exacerbations. However, all patients who had restarted *etanercept* responded satisfactorily.

It should be emphasized that biological treatment is used in patients with the most active course of the disease, not responding to methotrexate or other DMARDs. Probably, the majority (2/3) of these patients require a permanent or much longer *etanercept* therapy than quoted in this paper.

## Conclusions

Our study does not show correlation between the duration of remission off *etanercept* with duration of total *etanercept* treatment duration, clinical remission on *etanercept*, type of disease onset and duration of disease to introduction *etanercept*.

Discontinuation of *etanercept* therapy in a substantial proportion of JIA patients resulted in early exacerbation of the disease.

In many cases, reintroduction of *etanercept* is needed. Patients, in whom *etanercept* was restarted, responded satisfactorily.
